# Supplementary material for: Ophthalmic care at an academic medical centre for patients who were incarcerated or in immigration detention
Source: Eye (Lond). 2025 Sep 25;39(16):3015–20. doi: 10.1038/s41433-025-04005-8 (PMC12583815; doi:10.1038/s41433-025-04005-8)

**SUPPLEMENTARY INFORMATION**

**Supplementary Table 1**. Most Common Planned Surgeries and Procedures Performed between 11/2018-5/2023

| **Surgery/Procedure*** | **Count (n)** |
| --- | --- |
| Vitrectomy | 19 |
| Cataract Surgery | 12 |
| Glaucoma Drainage Device | 5 |
| Strabismus Surgery | 4 |
| Penetrating Keratoplasty | 4 |
| Nasolacrimal Duct Repair | 4 |
| Eyelid Surgery | 4 |
| Orbital Decompression | 4 |
| Scleral Buckle | 3 |
| Laser Peripheral Iridotomy | 2 |
| Selective Laser Trabeculoplasty | 2 |
| YAG Capsulotomy | 1 |

* Intravitreal injections were excluded as the decision to administer was often made the day of clinic.

**Supplementary Methods.** Visual Acuity and Intraocular Pressure Trends

The best corrected distance VA was reported per eye for each visit was converted from Snellen to logMAR. The mean and standard deviation for visual acuity recorded in the first visit, as well as for the last visit, were calculated, for all patients and for various subsets of patients, based on ophthalmic diagnoses that were reported during the study period: retinal detachments, cataracts, glaucoma, or orbital fractures. These calculations were also calculated for IOP for the first visit and last visit. Due to the non-normal distribution of these values, the Mann-Whitney U test was used to assess statistical differences, with p ≤ 0.05 thresholded for statistical significance.

The mean visual acuity (logMAR) at first and last visit recorded in the study interval was 0.5 ± 0.7 and 0.4 ± 0.7 respectively (p = 0.16) (**Supplementary Figure 1A**). The mean IOP at the first and last visit recorded in the study interval was 14.6 ± 5.0 and 14.4 ± 5.0 mmHg respectively (p = 0.35) (**Supplementary Figure 1B**). Subgroup analyses of VA and IOP by the most common diagnoses (retinal detachment, cataract, orbital wall fracture, and glaucoma) did not yield any statistically significant differences (**Supplementary Figures 2-5**, respectively).

**Supplementary Figure 1.** Visual acuity in LogMAR (A) and IOP (B) by eye between first and last visit for all patients

A)


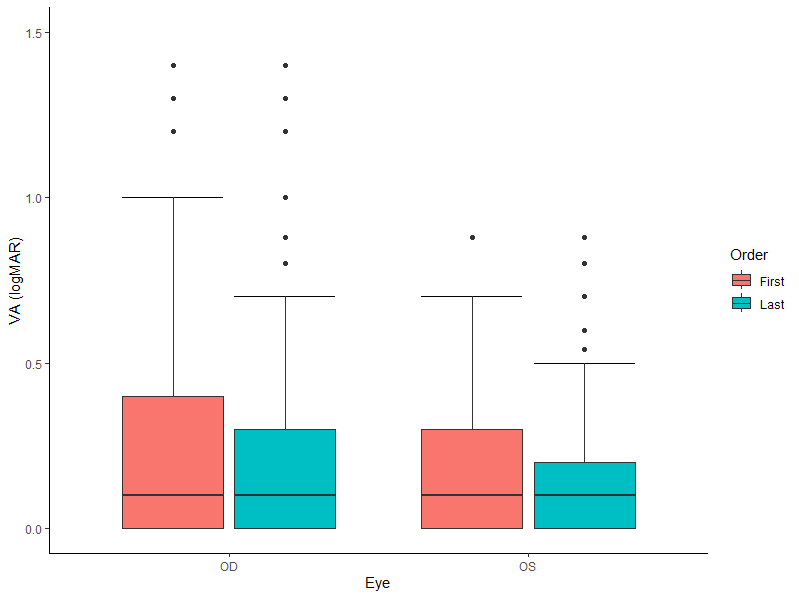


B)


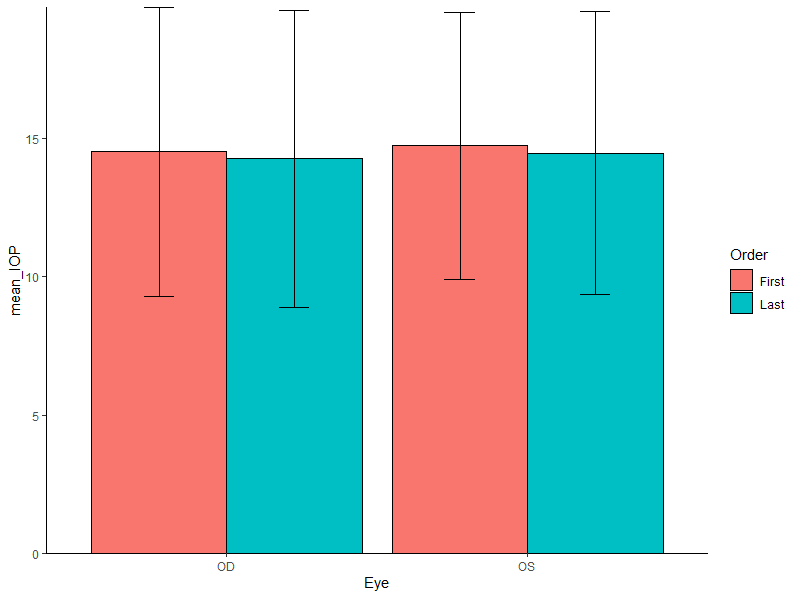


**Supplementary Figure 2.** Visual acuity in LogMAR (A) and IOP (B) by eye between first and last visit for all patients with retinal detachments

A)


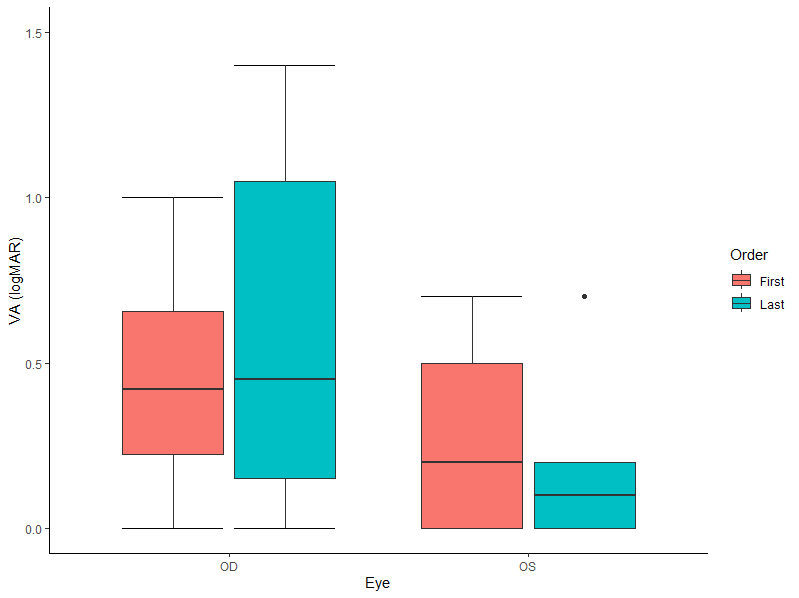


B)

**
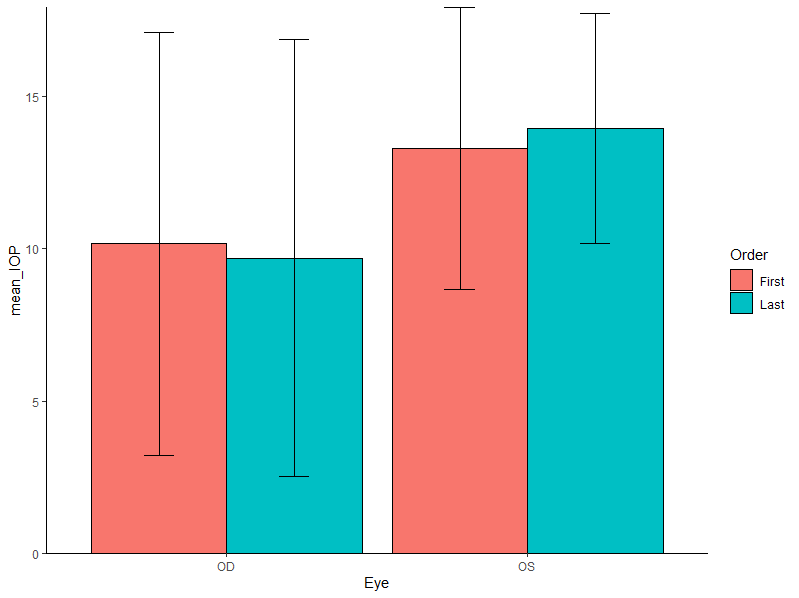
**

**Supplementary Figure 3.** Visual acuity in LogMAR (A) and IOP (B) by eye between first and last visit for all patients with cataracts

A)


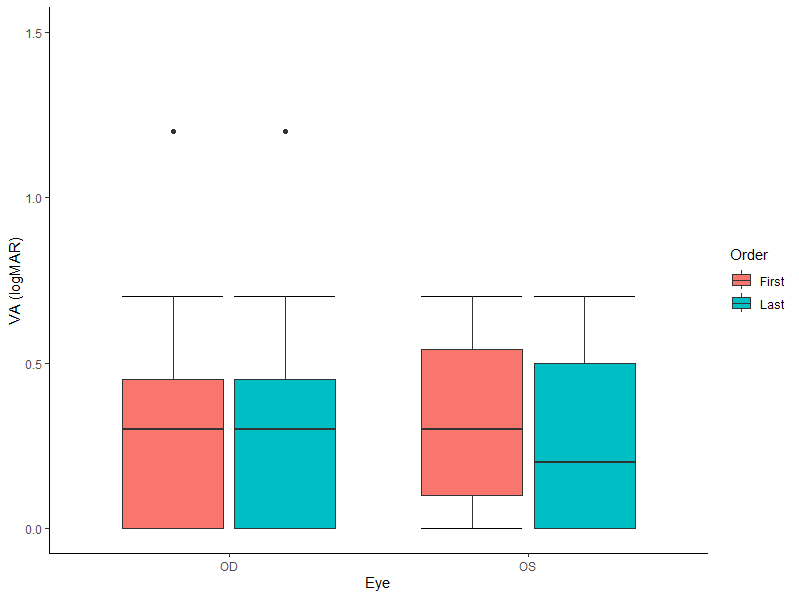


B)

**
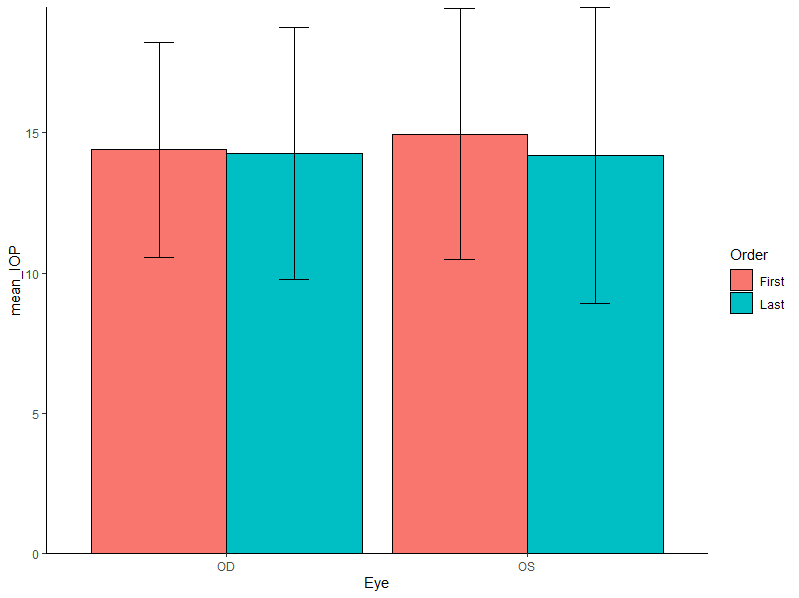
**

**Supplementary Figure 4.** Visual acuity in LogMAR (A) and IOP (B) by eye between first and last visit for all patients with glaucoma

A)


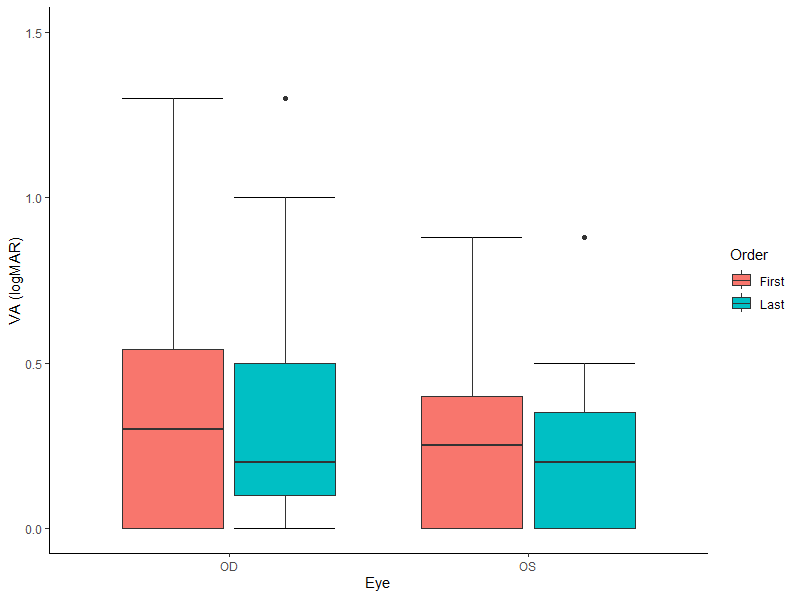


B)


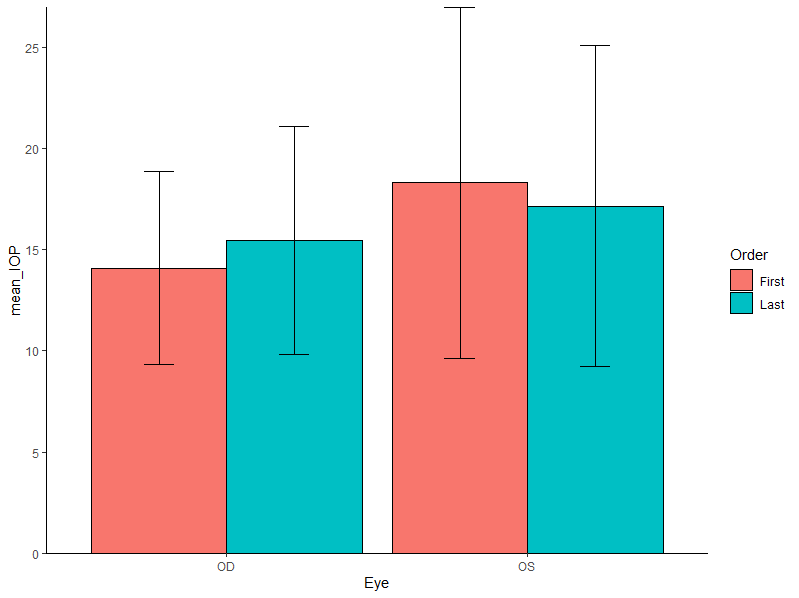


**Supplementary Figure 5.** Visual acuity in LogMAR (A) and IOP (B) by eye between first and last visit for all patients with orbital wall fractures

A)


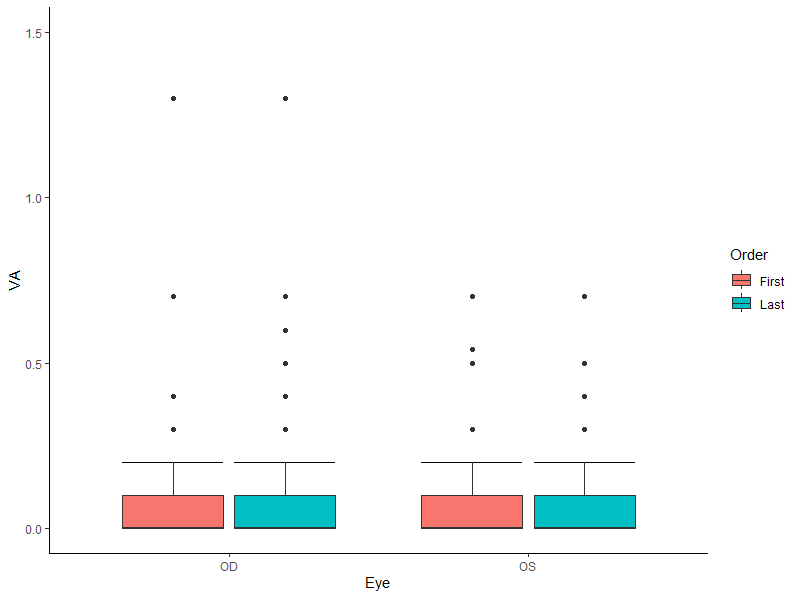


B)


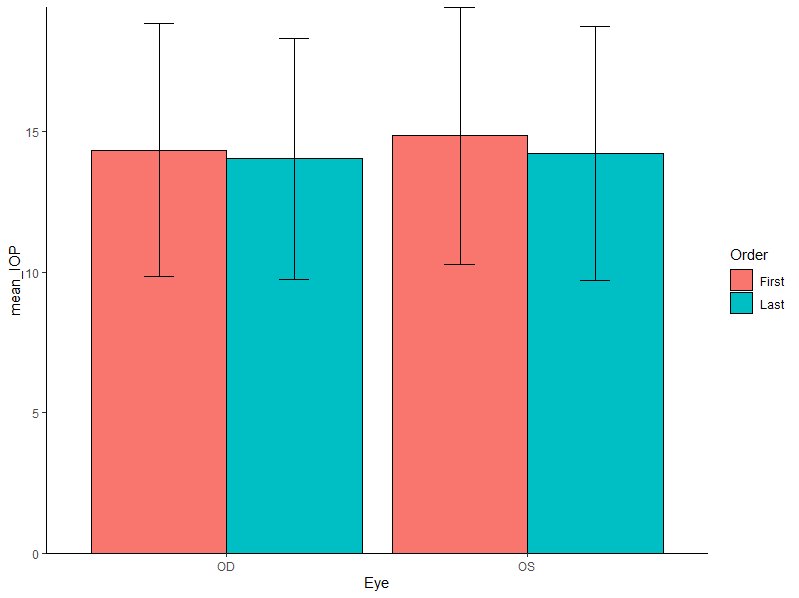

Supplement: Supplementary file 1 — SUPPLEMENTARY INFORMATION [file 41433_2025_4005_MOESM1_ESM.docx]
